# Schwannome cervical du nerf vague: Stratégies diagnostique et thérapeutique

**DOI:** 10.11604/pamj.2013.14.76.2325

**Published:** 2013-02-26

**Authors:** Najib Benmansour, Yasine Elfadl, Amal Bennani, Mustapha Maaroufi, Leila Chbani, Afaf Amarti, Siham Tizniti, Mohamed Noureddine Elalami

**Affiliations:** 1 Faculté de Médecine et de Pharmacie, Université Sidi Mohammed Benabdellah, Fès, Maroc; 2 Service d'ORL et CCF, CHU Hassan II, Fès, Maroc; 3 Service d'Anatomopathologie, CHU Hassan II, Fès, Maroc; 4 Service de Radiologie, CHU, Hassan II, Fès, Maroc

**Keywords:** Schwannome, nerf vague, scanner, imagerie par résonance magnétique, chirurgie, schwannoma, vagus nerve, scanner, magnetic resonance imaging, surgery

## Abstract

Les schwannomes cervicaux sont des tumeurs bénignes des nerfs périphériques développées exclusivement à partir des cellules de Schwann. L'atteinte du nerf vague cervical est relativement rare, et les auteurs en rappellent, à partir d'un cas, les signes radiologiques évocateurs ainsi que les caractéristiques histologiques. Le traitement de ces tumeurs est chirurgical. Un patient de 32 ans consultait pour une masse latéro-cervicale supérieure droite isolée, évoluant depuis trois ans. Une imagerie médicale (TDM et IRM) cervicale mettait en évidence une masse vascularisée au temps retardé, refoulant la veine jugulaire interne en dehors et l'axe carotidien en dedans. Un examen cytologique non contributif conduisait à réaliser une exérèse chirurgicale extracapsulaire de la masse par voie de cervicotomie. Il s'agissait d'une tumeur rétro-jugulo-carotidienne développée aux dépens du nerf vague cervical droit. L'analyse histologique concluait à un schwannome. Les suites opératoires étaient simples. Le schwannome du nerf vague est une tumeur bénigne rare, qui doit être évoquée devant toute masse latérocervicale isolée. L'imagerie médicale (TDM et IRM) cervicale préopératoire représente les examens de choix indispensable pour évoquer le diagnostic. Le traitement est chirurgical, afin de confirmer le diagnostic histologique. L'exérèse chirurgical complète extracapsulaire est possible et est le seul garant de la non récidive.

## Introduction

Le schwannome est une tumeur mésenchymateuse bénigne développée exclusivement à partir des cellules de la gaine de Schwann qui entourent les fibres nerveuses du système nerveux périphérique. Les schwannomes sont retrouvés dans 25% des cas au niveau cervical, le plus souvent à partir du nerf pneumogastrique (X) [1]. Ils se caractérisent par leur latence clinique et leur diagnostic difficile et tardif [1]. Le traitement de choix est chirurgical, mais l'abstention sera toujours discutée avec le patient qui sera informé des risques de séquelle fonctionnelle liés à cette chirurgie. À propos d'un cas récent de Schwannome du nerf vague cervical, nous proposons à travers une revue de la littérature une mise au point sur les aspects cliniques, radiologiques et thérapeutiques de cette entité anatomo-clinique rare.

## Patient et observation

**Figure 1:**
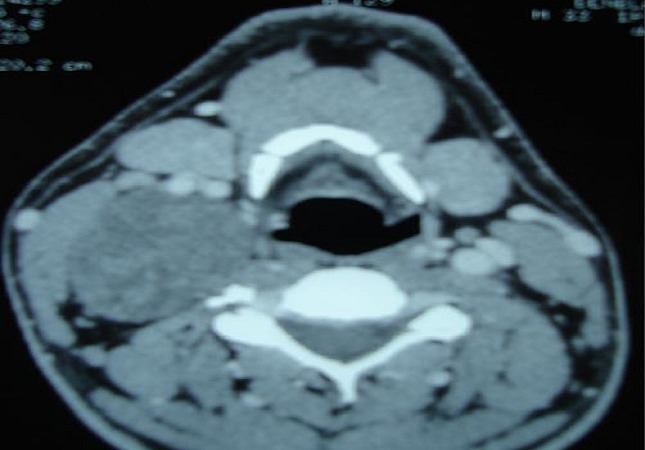
TDM cervicale avec injection en coupe axiale (A) et en coupe coronale (B): schwannome du nerf vague

**Figure 2:**
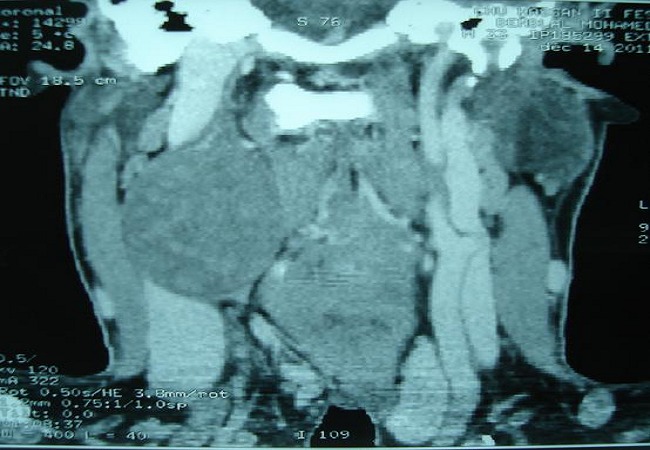
IRM cervico-faciale en coupe axiale séquence pondérée en T1 après injection de gadolinium: schwanome du nerf vague

**Figure 3:**
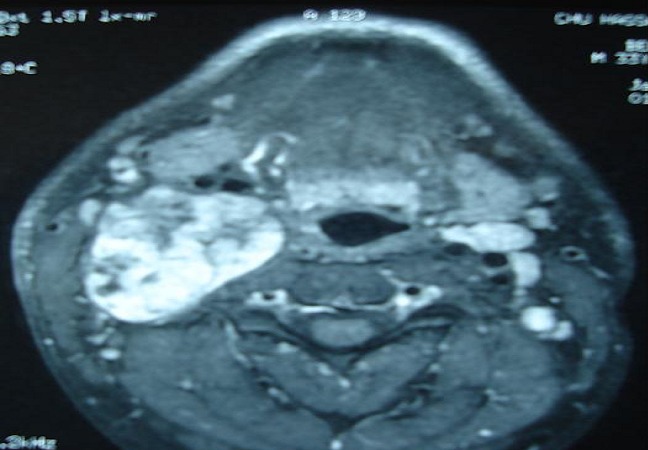
IRM cervico-faciale en coupe coronale avec injection: schwanome du nerf vague

**Figure 4:**
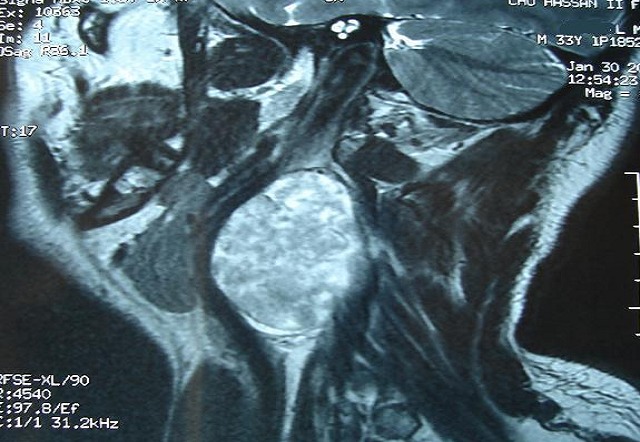
Vue peropératoire, schwannome du nerf vague droite

**Figure 5:**
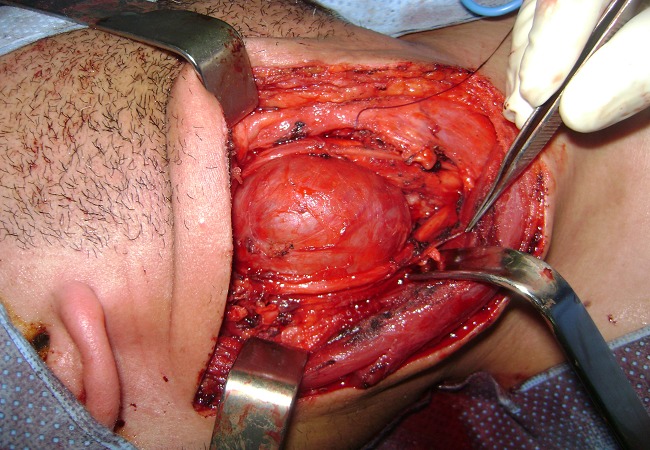
Shwannome du nerf vague. Nodules de Verocay constitués par des zones fibrillaires anucléés, bordées de part et d'autre d'une palissade de noyaux de type A d'Antoni. Coloration HES, grossissement x400

**Figure 6:**
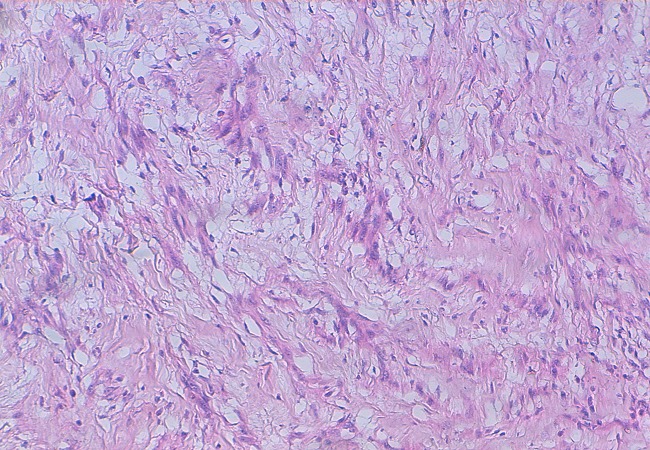
Aspect histologique schwannome, forte expression de la PS 100

Il s'agit d'un patient de 32 ans, sans antécédents pathologiques notables, consultait pour une masse latéro-cervicale droite, augmentant progressivement du volume depuis trois ans. L'examen physique retrouvait une masse isolée latéro-cervical supérieure droite de 6 x 3 cm, oblongue, ferme, insensible et sans signes inflammatoires en regard, mobile par rapport aux plans profonds et superficiels. Il n'y avait ni dysphonie, ni dysphagie, ni déficit neurologique. L'examen endobuccale montrait un refoulement en dedans de la région amygdalienne homolatérale. La tomodensitométrie cervicale (TDM) (Figure 1) mettait en évidence une masse ovoïde, bien limitée, de contours réguliers occupant les aires IIa et III droites, de densité tissulaire hétérogène en son centre, et mesurant 63 x 50 x 40 mm. Cette masse est située en dedans du muscle sterno-cléido-mastoïdien qui est refoulé ainsi la veine jugulaire interne qui est comprimée mais qui reste perméable. En dedans, elle refoule les vaisseaux carotidiens qui sont perméables. L'injection du produit de contraste montrait un rehaussement tardif de densité de la partie périphérique de la masse avec son centre qui restait isodense. L'IRM cervicale (Figure 2, Figure 3) montrait une volumineuse masse tissulaire interposée entre les vaisseaux jugulo-carotidiens et muscle sterno-cléido-mastoïdien, de forme grossièrement ovalaire, de contours polycycliques, présentant un hyposignal en T1, un hypersignal hétérogène en T2 et rehaussée de manière tardive et progressive après injection de gadolinium. Cette lésion refoule la carotide commune et sa bifurcation ainsi que la carotide interne et externe en avant, elle refoule la veine jugulaire interne latéralement qu'elle lamine sans l'obstruer totalement, elle refoule discrètement le carrefour pharyngo-laryngé sans retentissement significatif sur sa lumière. Elle mesure 58x52x37 mm dans ces plus grands diamètres. Le diagnostic d'un schwannome du vague a été évoqué. La cytoponction n'était pas contributive. L'exploration chirurgicale retrouvait une masse ovoïde homogène, bien encapsulée, refoulant en dehors la veine jugulaire interne et en dedans les vaisseaux carotidiens (Figure 4). La masse semblait être développée aux dépens du nerf vague. Une exérèse chirurgicale complète extracapsulaire préservant le nerf vague a été réalisée par voie de cervicotomie. L'examen anatomopathologique a objectivé une prolifération tumorale à cellularité modérée faite de cellules fusiformes à noyau ovoïde parfois allongé, à chromatine fine ou légèrement hétérogène. Certains noyaux sont volumineux polylobés, sans mitoses anormales. Ces cellules sont disposées en faisceaux courts enchevêtrés et en palissade formant des nodules Verocay. Ces nodules sont assez bien limités, parfois encapsulés évoquant un schwannome bénin de type A d'Antoni (Figure 5, Figure 6).

Les suites opératoires étaient simples. Aucune prise en charge complémentaire n'était proposée en dehors d'une simple surveillance. Le patient était revu à 6 mois et il se présentait totalement asymptomatique.

## Discussion

La première observation de schwannome cervical a été rapportée en 1899 par Ritter. Ces tumeurs surviennent au niveau de la tête et du cou dans 25 à 45% des cas [1, 2]. La localisation intracrânienne (nerf vestibulaire) est la plus fréquente [3]. La localisation cervicale est moins fréquente. Ces tumeurs peuvent se développer dans l'espace parapharyngé aux dépens des quatre derniers nerfs crâniens et du sympathique cervical [4–6].

Les schwannomes du nerf vague s′observent à tous âges avec une fréquence plus élevée chez l′adulte jeune. Le sex-ratio est égal pour la plupart des auteurs [1, 2]. Cependant, une prédominance féminine a été rapportée par certains auteurs [7]. La présentation clinique n'est pas pathognomonique. Il s'agit le plus souvent d'une masse latéro-cervicale isolée et asymptomatique, augmentant progressivement de volume [2, 4]. Cette masse peut, de par sa taille, entraîner une compression pharyngée et des signes peu spécifiques comme une gêne pharyngée, voire une odynophagie [2, 8]. Un Schwannome cervical du nerf vague est plus facilement évoqué quand la masse cervicale est associée à une dysphonie avec une paralysie de la corde vocale homolatérale. Cependant, ce dernier signe est rarement retrouvé au moment du diagnostic car dans ce cas, il est lié à la compression du nerf. En effet, contrairement au neurofibrome, le schwannome est une tumeur encapsulée qui n'infiltre jamais la gaine nerveuse périneurale et les filets nerveux [1, 4].

L'imagerie joue un rôle primordial dans la prise en charge de ces patients [1, 4, 6]. L'échographie est peu spécifique. La TDM et l'imagerie par résonance magnétique nucléaire permettent de préciser la taille de la tumeur, sa localisation parapharyngée dans le secteur II ou III cervical, son extension et ses rapports vasculaires avec les carotides interne et externe [6, 9]. En cas d'atteinte du X, la tumeur tend à élargir l'espace entre la carotide interne ou la carotide commune et la veine jugulaire interne, alors que l'atteinte du sympathique refoule vers l'avant l'axe jugulo-carotidien. Dans ces deux cas, la localisation est rétrostylienne.

La TDM élimine une adénopathie non vascularisée, un kyste congénital et un paragangliome du X ou du glomus carotidien, qui prend intensément le contraste dès le temps artériel de l'injection avec l'image classique d'une tumeur au-dessus et au contact de la bifurcation carotidienne élargissant l'espace entre les carotides interne et externe [1, 2, 10]. A la TDM, la tumeur est bien limitée et elle présente une densité inférieure à celle des muscles environnants. Après injection de produit de contraste, le rehaussement différé est en général homogène car la vascularisation dépend pour l'essentiel des capillaires intratumoraux. A l′IRM, en T1 la lésion est hypo ou isointense au muscle devenant hyperintense en T2, le rehaussement au Gadolinium est franc et intense [2, 7]. L'étude cytologique après cytoponction est souvent non contributive [2, 8]. Le diagnostic est anatomopathologique sur pièce chirurgicale, devant l'existence de cellules de schwann de morphologie et d'organisation différentes, classées selon Antoni en zone A où elles apparaissent en faisceaux, aux noyaux alignés en palissade et en zone B d'aspect kystique et de nature myxoïde. L'immunohistochimie montre l'expression prépondérante de la protéine S100 [1, 11].

Le traitement des schwannomes est chirurgical et le seul moyen de diagnostic. L'exérèse peut être complète du fait qu'ils sont bien encapsulés. La conservation du nerf d'origine est souvent possible car la tumeur a un développement extrinsèque par rapport à l'axe nerveux [7, 12]. Dans notre cas, lors de la cervicotomie, nous avons réalisé une exérèse complète extracapsulaire du schwannome préservant le nerf vague. La paralysie recurrentielle est alors la complication post-opératoire la plus fréquente en cas de traumatisme du X. L'utilisation d'un neuromonitoring préopératoire peut s'avérer utile dans la chirurgie des tumeurs nerveuses cervicales pour permettre le cas échéant d'envisager une résection tumorale partielle, par exemple en cas de diagnostic préopératoire de schwannome du X.

Les schwannomes cervicaux identifiés, de petite taille et de faible évolutivité, doivent faire rediscuter l'indication opératoire, en particulier ceux développés à partir du pneumogastrique, du grand hypoglosse ou d'une branche du plexus cervical [1].

Le pronostic des schwannomes est excellent. Les séquelles nerveuses sont exceptionnelles. La récidive locale est rare et est due probablement à une exérèse incomplète [1, 13]. La dégénérescence est exceptionnelle et même non admise car un schwannome serait d'emblée bénin ou malin.

## Conclusion

Le schwannome du nerf vague est une tumeur bénigne rare et de croissance lente, mais dont le volume important en l'absence de diagnostic cytologique doit faire discuter l'exploration chirurgicale. Une imagerie médicale (TDM et IRM) est indispensable dans le bilan préopératoire. L'exérèse chirurgicale complète extracapsulaire est possible et est le seul garent de la non récidive et de la préservation du nerf vague. Dans tous les cas, le patient doit être prévenu de la survenue postopératoire d'une paralysie recurrentielle pour laquelle il faut proposer une rééducation orthophonique et ou une médialisation de la corde vocale paralysée.
